# The Elusive Neuroendocrine Tumor: Finding the Ectopic ACTH Source 16 Years After the Diagnosis of Cushing Syndrome

**DOI:** 10.1210/jcemcr/luac035

**Published:** 2023-02-07

**Authors:** Galina Smushkin, Richard Phillips, Guillaume Chausse

**Affiliations:** Department of Medicine, Division of Endocrinology, University of British Columbia, Victoria, BC V6T 1Z3, Canada; Department of Medicine, Division of Endocrinology, University of British Columbia, Kelowna, BC V1V 1V7, Canada; Department of Radiology, McGill University, Montreal, QC H3G 1A4, Canada

**Keywords:** ACTH syndrome, ectopic, gastro-enteropancreatic neuroendocrine tumor, adrenalectomy, Cushing syndrome

## Abstract

Ectopic adrenocorticotropin hormone (ACTH) syndrome (EAS) accounts for the minority of cases of Cushing syndrome. Up to 20% of these cases remain occult, despite multiple imaging attempts to localize the ACTH-producing tumor. Here we describe long-term follow-up of a 41-year-old woman, with ectopic Cushing syndrome initially classified as occult due to negative localization studies, who had bilateral adrenalectomy to manage hypercortisolism. After 16 years and many computed tomography (CT) scans, magnetic resonance imaging scans, Octreoscans, and 2 exploration surgeries for false positives on imaging, the source of ectopic ACTH production was localized in the pancreas utilizing molecular imaging with gallium-68 somatostatin receptor–targeted positron emission tomography (PET)/CT and fluorine-18 fluorodeoxyglucose PET/CT. She underwent a distal pancreatectomy, and pathology confirmed a 1.7-cm well-differentiated pancreatic neuroendocrine tumor with a moderately strong reactivity to ACTH stain. This case demonstrates the utility of multiple functional imaging modalities in resolving these “cold cases” of occult ectopic Cushing syndrome and the importance of a timely management of hypercortisolism with bilateral adrenalectomy.

Ectopic adrenocorticotropin hormone (ACTH) syndrome (EAS) is responsible for 10% to 15% of Cushing syndrome cases. It is generally associated with a rapid progression of hypercortisolism, and a substantial morbidity and mortality. Inferior petrosal sinus sampling (IPSS) is the gold standard for differentiating a pituitary source of excess ACTH production from an ectopic source, the latter demonstrating an absence of a central-to-peripheral ACTH gradient. The diagnosis of EAS triggers imaging efforts to localize the ACTH-producing tumor. Tumors in the chest account for 70% of EAS cases, neuroendocrine tumors of the pancreas account for 10% to 15% of cases; other rare sources are medullary thyroid cancer, pheochromocytoma, other carcinoids, and rare carcinomas [1, 2]. High-resolution cross-sectional computed tomography (CT) imaging has a sensitivity of 50% to 67% to identify the source of ectopic ACTH production and when negative, a variety of nuclear medicine functional imaging techniques (Octreoscan, fluorine-18 fluorodeoxyglucose (18FDG) positron emission tomography/computed tomography PET/CT, and gallium-68 (68Ga) somatostatin receptor (SSTR)–targeted PET/CT) are employed [3]. Despite advances in these imaging modalities and an improving patient access, up to 20% of EAS remains occult after initial imaging and the clinician is faced with the challenge of managing hypercortisolism and organizing adequate imaging surveillance in an effort to identify the tumor over time. Few publications that addressed long-term follow-up of occult cases have reported negative results after a mean follow-up of 2 to 4 years [4, 5]. Here we present a case of a woman whose ACTH-producing pancreatic NET was found 16 years after the diagnosis with Cushing syndrome.

## Case Presentation

In 2001, a 41-year-old woman began experiencing leg edema, fatigue, frequent yeast infections, and weight gain. Over the next year, she developed facial plethora, proximal muscle weakness, purple striae, bruising, and hypertension.

### Diagnostic Assessment

In 2003, she was diagnosed with ACTH-dependent Cushing syndrome based on a 24-hour urine free cortisol 1165 nmol/day (0-193 nmol/day) (422 mcg/day) and plasma ACTH of 25 pmol/L (<10 pmol/L) (112.5 pg/mL). A magnetic resonance imaging (MRI) of the pituitary was normal, and an 8-mg overnight dexamethasone suppression test resulted in a morning cortisol of 275 nmol/L (10 mcg/dL) from a baseline morning cortisol of 589 nmol/L (21 mcg/dL). Notably, there is a lack of suppression of cortisol to less than 5 mcg/dL, yet there was suppression by more than 50% from baseline, highlighting the limited utility of this test. Inferior petrosal sinus sampling (IPSS) did not demonstrate a central-to-peripheral ACTH gradient (Table 1). A repeat 24-hour urine free cortisol in 2004 was 4675 nmol/day (50-220 nmol/day) (1695 mcg/day). She was treated with ketoconazole, as the search for the source of ectopic ACTH production began.

**Table 1.** Inferior petrosal sampling results

	Right IPS ACTH (pmol/L)	Left IPS ACTH (pmol/L)	Peripheral ACTH (pmol/L)
Baseline	28	30	27
1 minute post ovine CRH	31	31	27
3 minutes post ovine CRH	39	38	36
5 minutes post ovine CRH	53	54	51
10 minutes post ovine CRH	76	75	76

Abbreviations: ACTH, adrenocorticotropin hormone; CRH, corticotropin releasing hormone; IPS, inferior petrosal sampling.

Octreoscan showed a focus of activity in the right lower quadrant. A CT of the abdomen demonstrated bilateral complex cystic masses in her adnexa, right larger than left. The upper abdominal organs were described as unremarkable on the CT report. A carcinoid of the appendix was suspected, and the patient underwent a laparoscopic exploration and appendectomy. The appendix was normal on histopathology and the patient's hypercortisolism persisted. Postoperatively, a repeat Octreoscan showed no uptake. A high-resolution CT chest was unremarkable.

### Treatment

In 2006, the patient underwent laparoscopic bilateral adrenalectomy and had no complications. Histopathology showed bilateral compact cell hyperplasia. Within 6 months after the operation, glucocorticoids were tapered to physiologic doses and the symptoms of hypercortisolism resolved. On hydrocortisone replacement, 24-hour urine free cortisol was 62 nmol/day (24 mcg/day) with ACTH level of 55 pmol/L (250 pg/mL).

### Outcome and Follow-up

**Figure 1.**
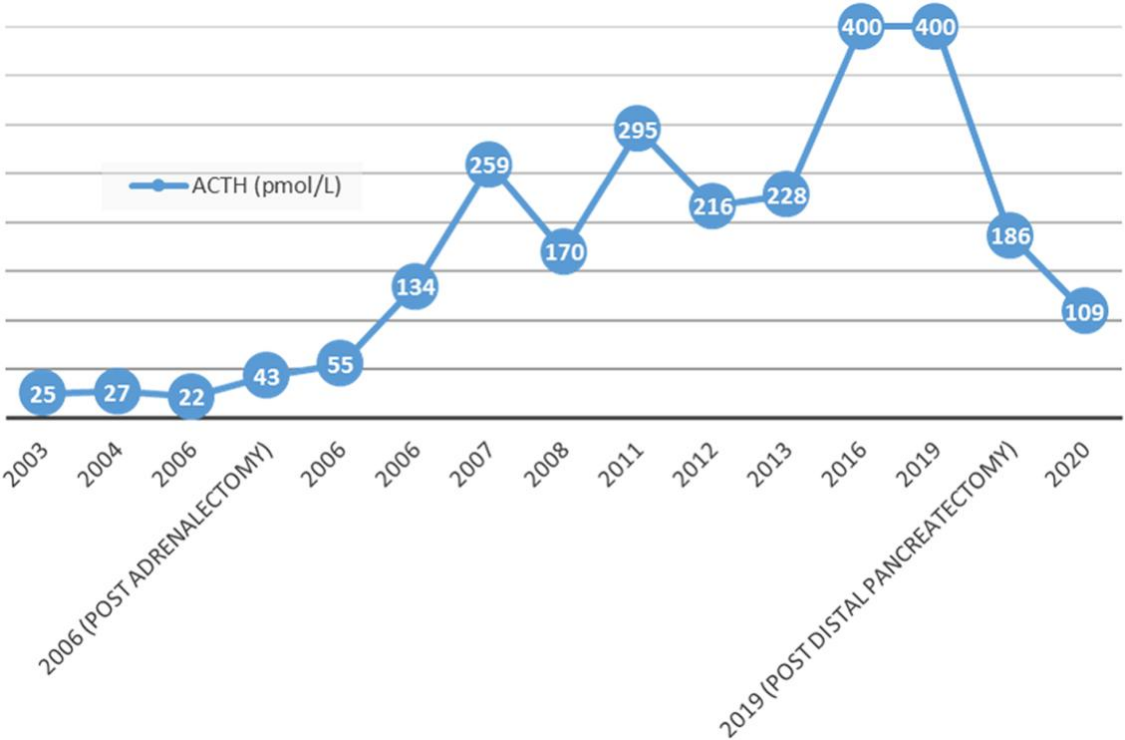
ACTH trends over time.

Between 2007 and 2019, the patient's ACTH levels rose (Fig. 1). She was compliant with hydrocortisone replacement, had no symptoms of adrenal insufficiency, but noted increasing skin hyperpigmentation. Administration of 8 mg dexamethasone did not suppress ACTH.

In 2011, she had bilateral salpingo-oophorectomy based on CT imaging reporting an adnexal mass. The pathology of the ovaries came back normal with several small functional cysts.

**Figure 2.**
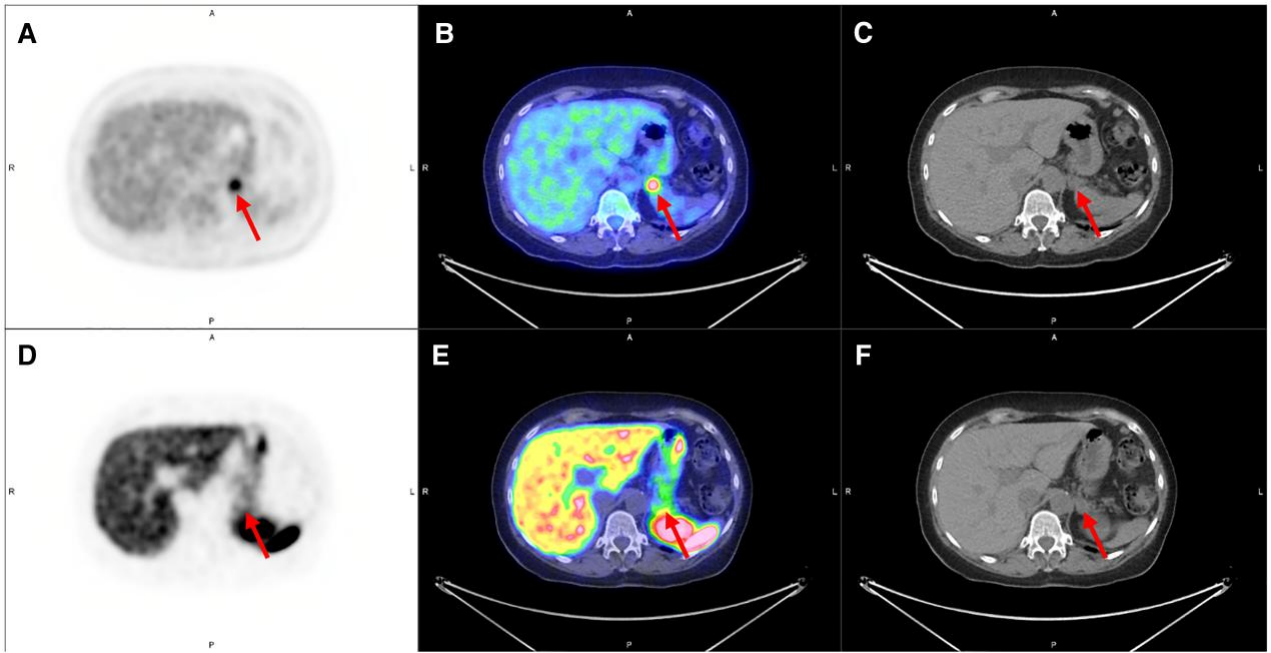
Select axial PET (A, D) CT (C, F) and PET/CT fusion (B, E) slices of a pancreatic tail lesion (arrow) with intense focal FDG uptake on [18F]FDG-PET/CT (A, B, C) and uncharacteristic absence of increased uptake on [68Ga]DOTATOC PET/CT (D, E, F) corresponding to a well-differentiated grade 1 pancreatic neuroendocrine tumor secreting ACTH.

**Figure 3.**
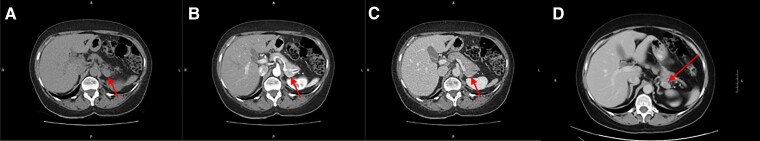
Select axial slices on triphasic CT abdomen of a subtle pancreatic tail lesion (arrow), with nearly the same attenuation as the neighboring pancreatic parenchyma on non-contrast (A), arterial phase (B), and venous phase (C) images. In retrospect, the same small lesion could be seen on a CT abdomen performed a decade prior (D), understandably misinterpreted as a pancreatic tail lobulation.

Between 2004 and 2019, she had 8 pituitary MRIs, 5 Octreoscans, and 6 CT scans. Early in her course, attempts were made to obtain access to FDG-PET scan but there were insurance barriers. On CT abdomen in 2018, note was made of a 1.7-cm soft tissue excrescence at the posterior tail of the pancreas, commented “likely reflects a lobule of pancreatic tissue.” In 2019, the patient underwent somatostatin receptor (SSTR)-targeted imaging (68Ga-DOTATOC PET/CT) and an 18FDG-PET/CT available through a research protocol. A pancreatic tail lesion was found with intense FDG uptake far exceeding the DOTATOC uptake, which was mild and similar to adjacent pancreas activity. As somatostatin analog activity reflects differentiated tissue and FDG activity characterizes more aggressive lesions, findings were concerning for a high-grade neuroendocrine tumor (NET) or an incidental pancreatic carcinoma (Fig. 2). At that point, a tri-phasic CT of the pancreas was performed, confirming a small subtle lobulation of tissue confluent with the posterior pancreatic tail, isoattenuating to the pancreas in all 3 phases (Fig. 3).

**Figure 4.**
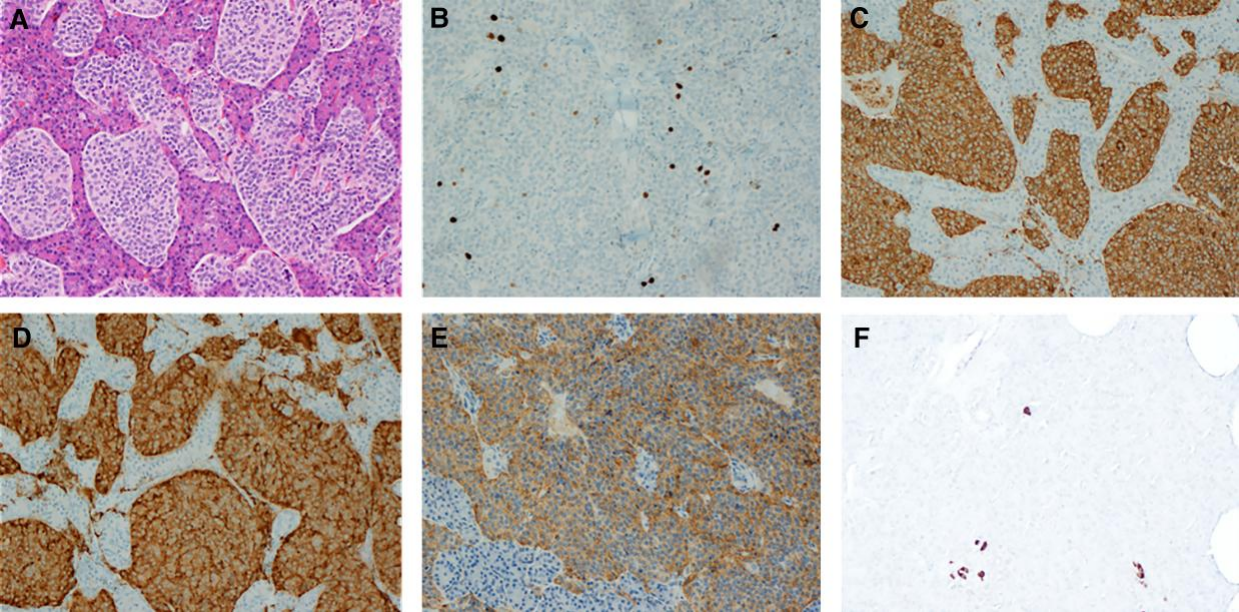
Well-differentiated pancreatic neuroendocrine tumor. (A) Hematoxylin and eosin staining, 10× magnification. (B) Immunohistochemical findings for Ki-67, 10× magnification. (C) Chromogranin positive. (D) Synaptophysin positive. (E) ACTH positive. (F) Somatostatin <1% of cells positive.

In 2019, 16 years after the diagnosis of ectopic Cushing syndrome, the patient underwent a distal pancreatectomy. Surgery and recovery were uneventful. Histopathology confirmed a 1.7-cm well-differentiated grade 1 pancreatic NET, (Ki67 index 1.5%) and moderately strong reactivity to ACTH on immunohistochemistry (Fig. 4). Postoperatively, ACTH decreased to 186.5 pmol/L (840 pg/mL). Her ACTH post 8 mg dexamethasone was 0.9 pmol/L (4 pg/mL). One year later, ACTH was 109 pmol/L (495 pg/mL), which is expected given bilateral adrenalectomy. The patient's skin hyperpigmentation resolved. Chromogranin A level was not measured preoperatively but was normal postoperatively.

## Discussion

This case illustrates several important challenges in the diagnosis and management of EAS. The results of IPSS were key in establishing the diagnosis, but after a decade of negative imaging, the clinicians considered the possibility that the IPSS results were false negative. The role of noninvasive diagnostic tests such as the peripheral corticotropin releasing hormone (CRH) and desmopressin tests has been studied in an effort to differentiate, in combination with imaging, Cushing disease from EAS without the use of IPSS [3]. Our patient's initial dynamic endocrine workup included only the high-dose dexamethasone suppression test, which has a limited diagnostic utility. Even if other noninvasive testing had pointed to EAS, negative initial cross-sectional imaging would have led to IPSS. Subsequent bilateral adrenalectomy precluded further noninvasive testing or repeating IPSS, as the ACTH response would be difficult to interpret given the baseline chronic ACTH elevation seen in these patients.

Although IPSS is the gold standard test for distinguishing Cushing disease from EAS, its specificity varies and is relatively lower than its sensitivity. False negative rates of 1% to 10% have been noted [6]. Anomalous petrosal venous drainage and variant venous anatomy can result in an absent ACTH gradient [7]. An analysis of diagnostic errors after IPSS by Swearingen et al. reported that the majority of negative IPSS are in fact false negatives, where the final diagnosis was Cushing disease due to a pituitary lesion, and only a minority of cases were true negatives, with a proven ectopic tumor [6]. Of note, in these series, all false negative responses after IPSS responded to CRH with a 35% peripheral ACTH level increase. Our patient's peripheral ACTH of 27 pmol/L (123 pg/mL) at baseline, increased to 76 pmol/L (345 pg/mL) 10 minutes post ovine CRH (Table 1), raising a suspicion for a false negative IPSS result. However, there are instances of false-positive response to CRH reported in patients with EAS [3]. Serial MRI imaging of the pituitary over time did not reveal any new lesion, whereas her peripheral ACTH level continued to rise (Fig. 1). The initial rise was attributed to adrenalectomy, but subsequent progression to a very high range, not responsive to high-dose dexamethasone suppression test, was more consistent with an ectopic source of ACTH production.

Over the years, our patient had multiple rounds of cross-sectional imaging which was reported as negative, and functional imaging was key in localizing her pancreatic NET in a surprising way. 68Ga-SSTR PET/CT is more sensitive than Octreoscan in detecting NETs, largely due to a higher affinity for SSTR type 2 than indium-111-Pentetreotide. Earlier literature reported a high sensitivity of 68Ga-SSTR PET in localization of ectopic Cushing syndrome at 81.8% to 100% [1]. However, a more recent systematic review has questioned those higher values in comparison to their own center series of 6 cases with 5 negative 68Ga-SSTR PET scans and 1 with decreased tracer uptake in a pancreatic NET [4]. Another retrospective case review of the use of this imaging modality in EAS in tertiary referral centers, found that 68Ga-DOTATATE identified 65% of primary tumors in EAS patients, the majority of whom had previous negative cross-sectional imaging [8]. Our patient's 68Ga-SSTR PET/CT scan showed an unimpressive amount of tracer uptake in the distal pancreas; it is the marked FDG uptake that led to the final diagnosis. Most guidelines on NETs do not indicate FDG-PET in their algorithm, as it generally detects tumors with higher proliferation activity. FDG-PET sensitivity in detecting the source of EAS is 52% [1]. However, in the systematic review of EAS in which 8.5% of tumors were pancreatic NETs, Isidori reported that 100% of pancreatic lesions were found by FDG-PET [1]. The head and the uncinate process of the pancreas demonstrate a physiologic uptake on 68Ga-SSTR PET due to the presence of somatostatin receptors on islet cells. Although physiologic uptake is usually associated with a lower standardized uptake value (SUV) range, one study of 68Ga-DOTA-NOC demonstrated a significant overlap of SUV values between physiologic uptake and tumors, concluding that focal intense uptake in the pancreas must be interpreted with caution and confirmation of tumor must be based on anatomic imaging [9]. In contrast, a recent case series of EAS where 56% of cases remained occult, did not report any instances of false-positive uptake in the pancreas on 68Ga-SSTR PET [5]. Similar to our case, Wannachalee et al reported that in retrospect an anatomic correlate to all DOTATATE-avid tumors could be identified on cross-sectional imaging [8]. This was our patient's first PET study, as she did not get access to this modality earlier in her workup due to insurance barriers. One can speculate that earlier access to PET would have eased management. This case remains puzzling in terms of molecular imaging as the final pathology of a well-differentiated pNET (Ki67 1.5%) was unexpected given high metabolic activity on FDG -PET and poor DOTATOC uptake. Pathology nevertheless matched her mostly indolent clinical course.

Management of hypercortisolism with bilateral adrenalectomy when the source of EAS was not identified was key to our patient's survival and good outcome. A recently published case series of ACTH-producing pNETs [10] describes 7 cases, where 3 had European Neuroendocrine Tumor Society stage I or II disease and the rest had either locally advanced or metastatic disease. Only 2 patients with early-stage disease achieved remission after pancreatic surgery. The 5 other patients died, the majority within 12 months of diagnosis, due to hypercortisolism-associated infections. One patient with early-stage disease but extremely high cortisol levels also died within a month of pNET enucleation, due to pulmonary infection and sepsis. In this series, only 2 patients underwent adrenalectomy. The authors concluded that a simultaneous bilateral adrenalectomy should be performed at the first surgery for rapid control of hypercortisolism.

## Learning Points

- The source of occult EAS can be identified more than a decade after the patient's initial diagnosis with Cushing syndrome and rounds of negative cross-sectional imaging, utilizing concurrent 68Ga-SSTR and FDG-PET.
- Timely management of severe hypercortisolism with bilateral adrenalectomy is essential for survival in cases of occult EAS.
- An increase in peripheral ACTH in response to CRH may suggest that a negative IPSS result is a false negative; however, a false-positive peripheral ACTH response can also be seen in EAS.
- Pancreatic ACTH-secreting tumors, mostly characterized as large and aggressive in literature, can be slow-growing and indolent.

## Data Availability

Data sharing is not applicable to this article as no datasets were generated or analyzed during the current study.

## Abbreviations

ACTH: adrenocorticotropin hormone
CRH: corticotropin releasing hormone
CT: computed tomography
EAS: ectopic ACTH syndrome
18FDG: fluorine-18 fluorodeoxyglucose
IPSS: inferior petrosal sinus sampling
MRI: magnetic resonance imaging
NET: neuroendocrine tumor
PET/CT: positron emission tomography/computed tomography
pNET: pancreatic neuroendocrine tumor
SSTR: somatostatin receptor

## References

[1] Isidori AM , Sbardella E, Zatelli MC, et al Conventional and nuclear medicine imaging in ectopic Cushing's syndrome: a systematic review. J Clin Endocrinol Metab. 2015;100(9):3231–3244. 26158607 10.1210/JC.2015-1589 PMC4570166

[2] Young J , Haissaguerre M, Viera-Pinto O, Chabre O, Baudin E, Tabarin A. Management of endocrine disease: Cushing's syndrome due to ectopic ACTH secretion: an expert operational opinion. Eur J Endocrinol. 2020;18(4):R29–R58. 10.1530/EJE-19-0877 31999619

[3] Frete C , Corcuff JB, Kuhn E, et al Non-invasive diagnostic strategy in ACTH-dependent Cushing's syndrome. J Clin Endocrinol Metab. 2020;105(10):3273–3284. 10.1210/clinem/dgaa409 32594169

[4] Varlamov E , Hinojosa-Amaya JM, Stack M, Fleseriu M. Diagnostic utility of gallium-68 somatostatin receptor PET/CT in ectopic ACTH-secreting tumors: a systematic literature review and single-center clinical experience. Pituitary. 2019;22(5):445–455. 31236798 10.1007/s11102-019-00972-w

[5] Zisser L , Kulterer A, Itariu B, et al Diagnostic role of PET/CT tracers in the detection and localization of tumours responsible for ectopic Cushing's syndrome. Anticancer Res. 2021;41(5):2477–2484. 33952474 10.21873/anticanres.15024

[6] Swearingen B , Katznelson L, Miller K, et al Diagnostic errors after inferior petrosal sinus sampling. J Clin Endocrinol Metab. 2004;89(8):3752–3763. 15292301 10.1210/jc.2003-032249

[7] Doppman JL , Chang R, Oldfield EH, Chrousos G, Stratakis CA, Nieman LK. The hypoplastic inferior petrosal sinus: a potential source of false-negative results in petrosal sampling for Cushing's disease. J Clin Endocrinol Metab. 1999;84(2):533–540. 10022412 10.1210/jcem.84.2.5475

[8] Wannachalee T , Turcu AF, Bancos I, et al The clinical impact of [68Ga]-DOTATATE PET/CT for the diagnosis and management of ectopic adrenocorticotropic hormone-secreting tumours. Clin Endocrinol. 2019;91(2):288–294. 10.1111/cen.14008 PMC6689243 31066920

[9] Krausz Y , Rubinstein R, Appelbaum L, et al Ga-68 DOTA-NOC uptake in the pancreas. Clin Nuclear Med. 2012;37(1):57–62. 10.1097/RLU.0b013e3182393404 22157030

[10] Zhang C , Jin J, Jing X, et al The clinical features and molecular mechanisms of ACTH-secreting pancreatic neuroendocrine tumours. J Clin Endocrinol Metab. 2020;105(11):3449–3458. 10.1210/clinem/dgaa507 32785672

